# Prediction of Week 4 Virological Response in Hepatitis C for Making Decision on Triple Therapy: The Optim Study

**DOI:** 10.1371/journal.pone.0122613

**Published:** 2015-03-31

**Authors:** Manuel Romero-Gómez, Juan Turnes, Javier Ampuero, Itziar Oyagüez, Beatriz Cuenca, Juan Gonzalez-Garcia, Belén Muñoz-Molina, Rocio Aguilar, Sandra Leal, Ramon Planas, Javier Garcia-Samaniego, Moises Diago, Javier Crespo, Jose Luis Calleja, Miguel Angel Casado, Ricard Sola

**Affiliations:** 1 UCM Digestive Diseases & ciberehd, Valme University Hospital, Sevilla, Spain; 2 Complejo Hospitalario de Pontevedra, Pontevedra, Spain; 3 Pharmacoeconomics & Outcomes Research Iberia, Madrid, Spain; 4 Hospital Infanta Cristina, Parla, Madrid, Spain; 5 Hospital Universitario La Paz, Madrid, Spain; 6 Unidad de Hepatología, Roche Farma, Spain; 7 FISEVI, Sevilla, Spain; 8 Hospital Germans Trias i Pujol & ciberehd, Badalona, Barcelona, Spain; 9 Hospital Carlos III & ciberehd, Madrid, Spain; 10 Hospital General de Valencia, Valencia, Spain; 11 Hospital Marqués de Valdecilla, Santander, Spain; 12 Hospital Puerta de Hierro, Madrid, Spain; 13 Hospital del Mar, IMIM, Barcelona, Spain; University of Sydney, AUSTRALIA

## Abstract

**Background:**

Virological response to peginterferon + ribavirin (P+R) at week 4 can predict sustained virological response (SVR). While patients with rapid virological response (RVR) do not require triple therapy, patients with a decline <1log_10_ IU/ml HCVRNA (D1L) should have treatment discontinued due to low SVR rate.

**Aim:**

To develop a tool to predict first 4 weeks’ viral response in patients with hepatitis C genotype 1&4 treated with P+R.

**Methods:**

In this prospective and multicenter study, HCV mono-infected (n=538) and HCV/HIV co-infected (n=186) patients were included. To develop and validate a prognostic tool to detect RVR and D1L, we segregated the patients as an estimation cohort (to construct the model) and a validation cohort (to validate the model).

**Results:**

D1L was reached in 509 (80.2%) and RVR in 148 (22.5%) patients. Multivariate analyses demonstrated that HIV co-infection, Forns’ index, LVL, IL28B-CC and Genotype-1 were independently related to RVR as well as D1L. Diagnostic accuracy (AUROC) for D1L was: 0.81 (95%CI: 0.76 — 0.86) in the estimation cohort and 0.71 (95%CI: 0.62 — 0.79) in the validation cohort; RVR prediction: AUROC 0.83 (95%CI: 0.78 — 0.88) in the estimation cohort and 0.82 (95%CI: 0.76 — 0.88) in the validation cohort. Cost-analysis of standard 48-week treatment indicated a saving of 30.3% if the prognostic tool is implemented.

**Conclusions:**

The combination of genetic (IL28B polymorphism) and viral genotype together with viral load, HIV co-infection and fibrosis stage defined a tool able to predict RVR and D1L at week 4. Using this tool would be a cost-saving strategy compared to universal triple therapy for hepatitis C.

## Introduction

Hepatitis C virus (HCV) infection affects up to 150 million people worldwide, and is a major cause of liver cirrhosis and hepatocellular carcinoma. HCV is classified into six genotypes, with genotype 1 being the predominant genotype in Europe. The classical definition of non-response to peginterferon + ribavirin (P+R) treatment in genotype 1 is HCV-RNA decline at week 12 to <2 log_10_, or positive viral load at week 24 [1]. Further, a decline at week 4 of treatment of 1 log_10_ HCV-RNA (D1L) may be used in scheduling patients to receive protease inhibitor-based triple therapy [2]. Genotype IL28B rs12979860 has shown a strong association with sustained viral response (SVR) in mono-infected HCV [3], and in HCV/HIV co-infected patients [4]. Also, IL28B correlates with a faster 1^st^ and 2^nd^ phase decline in viral load during treatment, and a more rapid virological response (RVR) rate [5]. Metabolic factors such as insulin resistance (measured as the HOMA index) and fibrosis were also strongly related to SVR [6]. In patients receiving boceprevir-based triple therapy, virological response at week 4 following double P+R therapy could predict SVR; patients without D1L at week 4 showing a very low SVR rate (around 13%) despite protease inhibitor being added [7]. Conversely, patients achieving RVR showed a very high SVR rate with double as well as with triple therapy [8]. Hence, predicting the response to P+R at week 4 would facilitate the decision of whether or not to add a protease inhibitor to the treatment (which would not be necessary in RVR patients) and such a treatment could be avoided in patients without D1L.

The primary aim of the current study was to develop, and validate, a prognostic tool to predict RVR and D1L at week 4 in patients with HCV genotypes 1 & 4 treated with peginterferon-α-2a + ribavirin. The secondary aim was to assess the economic impact for the different strategies using this proposed “Optim” tool.

## Materials and Methods

**Fig 1:**
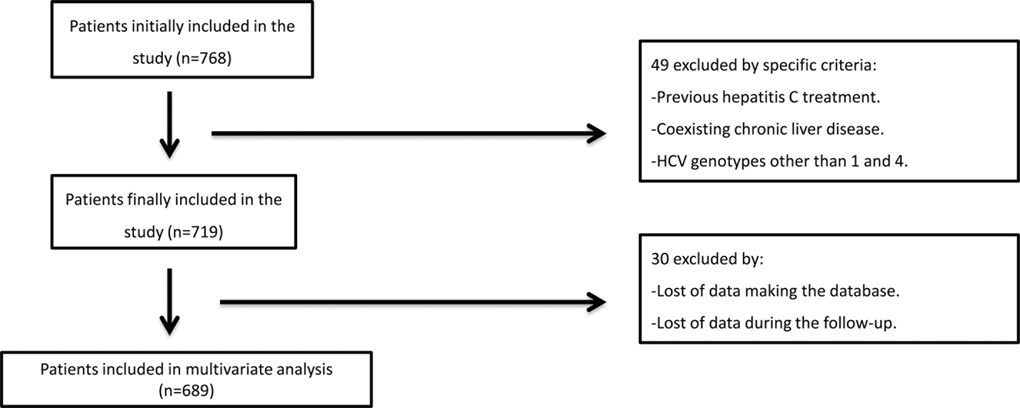
Flow-chart of the study.

HCV-infected patients (n = 768) were recruited into this prospective, multicenter study (ClinicalTrials.gov ID NCT01884402). Enrollment in the 83 participating Spanish hospitals was between the years 2010 and 2012. All patients provided written informed consent to participate in the study and for the collection and storage of peripheral blood mononuclear cells for host DNA and IL28B analysis. The study was approved by the Spanish Committee for Post-authorization Studies and by the CEIC of Valme University Hospital, the CEIC of Complejo Hospitalario de Pontevedra, the CEIC of Hospital Infanta Cristina, the CEIC of Hospital Universitario La Paz, the CEIC of Hospital Germans Trias i Pujol, the CEIC of Hospital Carlos III, the CEIC of Hospital General de Valencia, the CEIC of Hospital Marques de Valdecilla, the CEIC of Hospital Puerta de Hierro and the CEIC of Hospital del Mar. The study was conducted in accordance with the recommendations of the Declaration of Helsinki and good Clinical Practice Guidelines. All patients with HCV-genotype 1 and 4 followed standard treatment of peginterferon alpha-2a and ribavirin for 48 weeks. Patients received peginterferon α-2a (Pegasys; Roche 180μ/week) combined with ribavirin 1,000 mg/day if body weight was ≤75kg, or 1,200mg if body weight was >75 kg. Peginterferon and ribavirin dose modification were according to standard criteria and procedures [9]. Inclusion criteria were: patients >18 years of age diagnosed as having chronic hepatitis C with HCV-RNA positivity; who had criteria for commencing antiviral therapy in clinical practice; classified as genotype 1 or 4 whether or not co-infected with HIV. Exclusion criteria were: previous treatment with P+R; any coexisting chronic liver disease (including HBV infection); HCV genotypes other than 1&4 (i.e. types 2, 3, 5 and 6) (Fig 1). At baseline, all patients had a quantitative measurement of serum or plasma HCV-RNA performed using the polymerase chain reaction (PCR) assay with the COBAS AmpliPrep/COBAS TaqMan HCV Test (Roche Diagnostics GMBH, Mannheim). RVR was defined as undetectable serum HCV-RNA using a sensitive qualitative assay (lower limit of detection of 15 IU/mL). A qualitative measurement of serum HCV-RNA was performed at weeks 4, 8, 12, 24, 48 of treatment, and at weeks 4, 12, and 24 during follow-up. High viral load (HVL) of HCV was defined as ≥800,000 IU/L. HCV genotyping was performed by reverse hybridization (Versant HCV® 2.0 Assay LiPA, Siemens, Uppsala, Sweden) in all patients. IL28B genotyping was performed using real-time PCR (RT-PCR) using the LightCycler 480 System (Roche Diagnostic). The SNP rs12979860 is located 3kb upstream of the IL28B gene on chromosome 19. Fibrosis stage was defined using non-invasive methods: a) the aspartate aminotransferase-to-platelet ratio index (APRI) calculated as [(AST/upper limit of normal laboratory reference range) x 100] / platelet count 10^9^/L) with proposed cutoff values of >1.5 and >2 to bridging fibrosis and cirrhosis, respectively [10]; b) fibrosis 4 score (FIB-4) calculated as [(age x AST (IU/L)] / [platelet count (10^9^/L) x ALT (IU/L)1/2)] with proposed cutoff values of <1.45 for F0-F1 and >3.25 for F3-F4 [11]; and c) Forns’ index (calculated as (7.811–3.131 x ln [platelet count (10^9^/L)] + 0.781 x ln [GGT(IU/L)] + 3.467 x ln [age]– 0.014 [cholesterol (mg/dL)]) with proposed cutoff values of <4.2 for mild fibrosis and >6.9 for severe fibrosis [12]. Significant fibrosis was defined as FIB-4 >1.45 or Forns’ index >4.2. A total of 153 (21.1%) patients underwent liver biopsy before commencing therapy. Histologic evaluation was performed throughout the study by the same pathologist at each hospital, using Scheuer scoring [13]: F0 (no portal fibrosis), F1 (some portal fibrosis), F2 (slight bridging fibrosis), F3 (considerable bridging fibrosis), F4 (cirrhosis).

Statistical analyses included the Mann-Whitney U test, the Student *t*-test, or ANOVA for continuous variables, with the χ^2^ or the Fisher exact probability test for categorical data. Backward logistic regression was used in the multivariate analysis, in which only variables associated with the outcome in univariate analysis were included. Quantitative values are presented as means ± SD, median and quartiles Q1 and Q3, while qualitative values are presented as absolute and relative frequency. A probability value of p<0.05 was considered statistically significant. Statistical analyses were performed using SPSS version 20.0 software (SPSS, Chicago, IL, USA). To develop and validate a prognostic tool to detect RVR and D1L, we segregated the patients as an estimation cohort (to construct the model) and a validation cohort (to validate the model). Subsequently, the overall cohort was stratified by co-infection, HCV genotype, IL28B polymorphism and viral load (689 patients had all these 4 variables recorded). Patients were, then, randomized 2:1 as estimation cohort (66.6%; 458/689) and as validation cohort (33.3%; 231/689). Estimation and validation cohort were well matched, according to age (45.7 ± 9.2 *vs*. 46.3 ± 8.3), gender distribution (male sex 65.7% *vs*. 66.2%), IL28B polymorphism (CC genotype 37.1% *vs*. 38.5%) and viral genotype (HCV-1 79.9% *vs*. 83.5%).

A cost-analysis was performed to assess the cost differences of 48 weeks of treatment, with or without implementation of the prognostic tool. A decision tree was designed based on the sensitivity of the prognostic tool to predict RVR and D1L. Time horizon was <1 year and, therefore, no discount rate was applied.

Pharmaceutical costs were calculated according to the Summary Product Characteristics, and assuming the whole recommended duration. Rules for treatment cessation were not considered for any of the therapies. Mandatory rebate was applied to ex-factory prices for boceprevir, telaprevir and peginterferon, and ex-factory price for generic ribavirin was used. Triple therapy cost was calculated as the average cost of boceprevir and telaprevir treatments for 48 weeks (€35,233). A viral load determination at week 4 was applied only to those patients in whom RVR was expected following the implementation of prognostic tool to predict RVR. The unit cost per determination (€121, at 2013 prices) was obtained from a National Health-cost database (*eSalud*) [14]. Alternative scenarios were tested by modifying the sensitivity and positive predictive value of the prognostic tools (with 95%CI limits), and triple therapy cost.

## Results

### Baseline characteristics

Baseline epidemiological and biochemical features of the overall cohort are shown in Table 1. Gender distribution was 66.5% (478/719) males and 33.5% (241/719) females. Mean age was 45.9 ± 8.9 years of age. Mono-infected patients represented 74.8% (538/719), while 25.2% (181/719) were co-infected. HCV genotype distributions were: 80.9% (582/719) genotype 1 and 19.1% (137/719) genotype 4. IL28B polymorphism was CC in 37.7% (260/690) and CT/TT in 62.3% (430/690). Basal high HCV-viral load (HVL) was present in 59.1% (424/718). Fibrosis stage was measured by liver biopsy in 153 patients and the stages scored as: 19.6% (30/153) F0; 33.3% (51/153) F1; 27.5% (42/153) F2; 12.4% (19/153) F3; and 7.4% (11/153) F4. With non-invasive methods, significant fibrosis was 44.9% (311/693), and 16.9% (113/669) were cirrhotic. Insulin resistance (HOMA index ≥ 2) was noted in 64.6% (267/413) of patients.

**Table 1:** Baseline characteristics of the overall patient population.

Characteristic		N
Gender distribution; males		66.5% (478/719)
Age; years ± SD		45.9 ± 8.9
Mono-infected patients; HCV		74.8% (538/719)
	HCV genotype 1	80.9% (582/719)
	HCV genotype 4	19.1% (137/719)
Co-infected patients; HCV + HIV		25.2% (181/719)
IL28B polymorphism		
	CC	37.7% (260/690)
	CT/TT	62.3% (430/690)
HVL		59.1% (424/718)
Fibrosis; liver biopsy		
	F0	19.6% (30/153)
	F1	33.3% (51/153)
	F2	27.5% (42/153)
	F3	12.4% (19/153)
	F4	7.2% (11/153)
Fibrosis; non-invasive methods		44.9% (311/693)
	FIB-4	2± 2.3
	APRI	1.1 ± 1.5
	Forns’ index	5.5 ± 2
Insulin resistance; HOMA-IR > 2		64.6% (267/413)

We found the following variables significantly different when comparing mono-infected *vs*. co-infected patients (Table 2): age, gender distribution, BMI, HCV genotype distribution, ALT, platelet count, triglycerides, significant fibrosis, number of cirrhotic patients and HVL.

**Table 2:** Comparison of baseline characteristics; mono-infected *vs*. co-infected patients.

Characteristic	Mono-infected	Co-infected	p
Gender distribution; male	61.9% (331/534)	79.4% (147/185)	<0.001
Age; years ± SD	46.4 ± 9.7	44.6 ± 6.1	<0.005
HCV genotype 1	85% (453/533)	69% (129/186)	<0.001
HCV genotype 4	15% (80/533)	31% (57/186)	<0.001
IL28B-CC polymorphism	38.2% (319/516)	36.2% (63/174)	0.652
BMI; kg÷m^2^ ± SD	26.6 ± 4.4	24.3 ± 4	<0.001
AST; IU/L ± SD	65.4 ± 53.4	58.2 ± 43	0.608
ALT; IU/L ± SD	95.7 ± 88.8	67.9 ± 52.8	<0.001
AST/ALT ± SD	0.8 ± 0.3	0.9 ± 0.4	<0.001
GGT; IU/L ± SD	89.2 ± 103.5	159.5 ± 182.6	<0.001
Hemoglobin; g/L ± SD	15 ± 1.5	15.1 ± 1.4	0.471
Platelet count; x10^9^/L ± SD	203.5 ± 67.6	178.3 ± 65.2	<0.001
Cholesterol; mg/dL ± SD	177.3 ± 37.7	176.8 ± 35	0.875
Triglycerides; mg/dL ± SD	108 ± 58.5	155.2 ± 78.5	<0.001
LDH (IU/L) ± SD	259 ± 109.1	249 ± 107.1	0.385
Glucose; mg/dL ± SD	99.9 ± 26	94.9 ± 14.3	0.138
Insulin; μU/mL ± SD	13.5 ± 10.8	13.6 ± 9.6	0.779
Insulin; resistance (HOMA-IR > 2)	64.9% (211/325)	63.6% (56/88)	0.900
FIB-4 ± SD	2 ± 2.1	2.3 ± 2.7	<0.005
APRI ± SD	1.1 ± 1.4	1.2 ± 1.6	0.129
Forns`index ± SD	5.2 ± 2	6.1 ± 1.8	<0.001
Significant fibrosis	41.6% (215/517)	54.5% (96/176)	<0.005
Cirrhotic patients	15% (74/493)	22% (39/177)	<0.05
High viral load	56.6% (301/532)	66.1% (123/186)	<0.05

### Prognostic factors related to RVR

**Fig 2:**
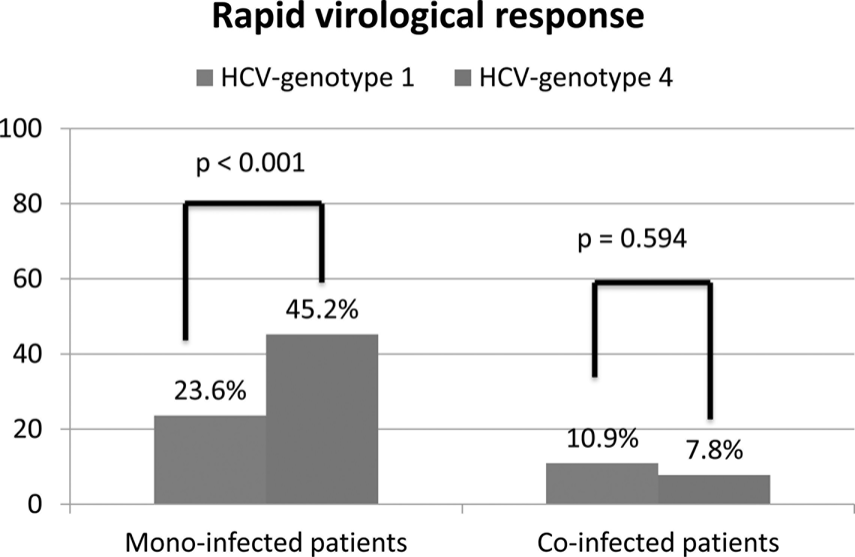
Rapid virological response in patients segregated with respect to mono-infection or co-infection

RVR was achieved in 22.5% (148/659) of the overall cohort. Age was lower in patients with RVR (43.3 ± 8.9 *vs*. 46.7 ± 8.6; p < 0.001). Co-infected patients showed a lower prevalence of RVR than mono-infected patients [10% (17/170) *vs*. 26.8% (131/489); p < 0.001]. RVR was higher in HCV genotype 4 than in HCV genotype 1 [29.8% (37/124) *vs*. 20.7% (111/535); p < 0.05]. Thus, HCV genotype 4 reached RVR in 45.2% (33/73) *vs*. 23.6% (98/416) in HCV genotype 1 (p < 0.001) in mono-infected patients (Fig 2). IL28B-CC showed RVR in 38% (90/237) of patients while the IL28B-CT/TT showed 13.2% (53/402) RVR response (p < 0.001). Insulin resistance was associated with lower RVR [19% (47/248) *vs*. 28.8% (38/132); p < 0.05)]. Conversely, cirrhotic patients had less RVR than non-cirrhotic patients [12.9% (13/101) *vs*. 24.1% (123/511; p < 0.05]. HVL was related to less RVR [13.1% (51/388) *vs*. 35.9% (97/270); p < 0.001]. FIB-4, APRI and Forns’ index were significantly decreased (p < 0.001) in patients with RVR.

### Development of the prognostic tool (“Optim”) to predict RVR

**Fig 3:**
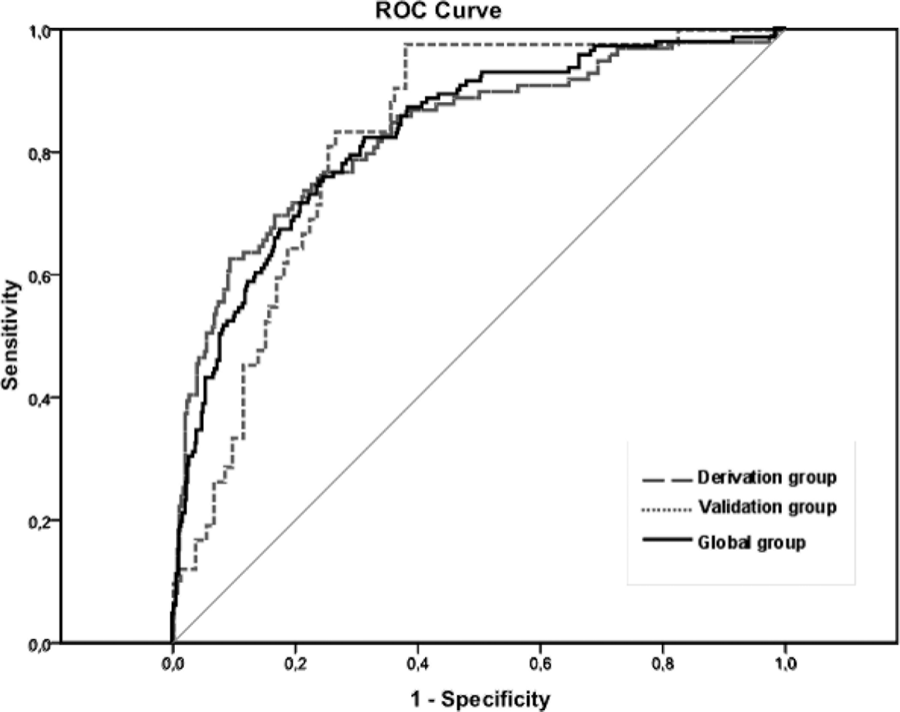
AUROC curve analysis for R_RVR_, including the overall cohort (0.82; p<0.001), the estimation cohort (0.83; p<0.001) and the validation cohort (0.82; p<0.001)

In multivariate analyses, variables associated with RVR were: LVL [Odds Ratio: 4.54 (95%CI: 2.47–8.34); p < 0.001], HIV co-infection [OR: 0.45 (95%CI: 0.22–0.91); p = 0.027], IL28B-CC [OR: 7.81 (95%CI: 4.29–14.38); p < 0.001], Genotype 1 [OR: 0.42 (95%CI: 0.21–0.81); p = 0.01], Forns’ Index [OR: 0.71 (95%CI: 0.60–0.83); p < 0.001] (Table 3). The AUROC for R_RVR_ was 0.83 (95%CI 0.79–0.87; p < 0.001) and the cut-off 0.248 showed a sensitivity of 76%, specificity of 75%, positive predictive value of 47% and negative predictive value of 91%. This model was confirmed in the validation cohort, except for the HCV genotype., with an AUROC of 0.82 (95%CI: 0.76–0.88; p < 0.001) (Fig 3) and a cut-off 0.248 showing a sensitivity of 69%, specificity of 77%, positive predictive value of 43% and negative predictive value of 91%.

**Table 3:** Variables associated with RVR in multivariate analysis of the estimation cohort.

Characteristics	O.R. (95% CI)	p
Co-infected patients	0.45 (95%CI: 0.22–0.91)	0.027
HCV genotype 1	0.42 (95%CI: 0.21–0.81)	0.01
IL28B-CC polymorphism	7.81 (95%CI: 4.29–14.38)	< 0.001
Forns`index	0.71 (95%CI: 0.60–0.83)	< 0.001
Low viral load	4.54 (95%CI: 2.47–8.34)	< 0.001

The final formula derived was:

$${\text{R}}_{\text{RVR}}\text{= 1 / }\left({\text{1+e}}^{\text{-(0.495 +1.513 x HVL – 0.797 x co-infection + 2.061 IL28B -0.873 x HCV genotype -0.345 x Forns’ index)}}\right)$$

Additionally, we obtained the AUROC in mono-infected HCV genotype 1 patients for R_RVR._ The AUROC for R_RVR_ was 0.79 (95%CI 0.73–0.86; p = 0.0001) in the estimation cohort and was 0.77 (95%CI 0.68–0.85; p = 0.0001) in the validation cohort.

The final formula derived was:

$$\text{Rrvr= 1/}\left({\text{1+e-}}^{\text{(-3.582 +1,202 x HVL + 2.258 IL28B + 1.188 x Forn`s index)}}\right)$$

### Prognostic factors related to D1L

D1L was reached in 80.2% (509/635) of the overall cohort. Co-infected patients showed a lower prevalence of D1L than mono-infected patients [70.2% (118/168) *vs*. 83.7% (391/467); p < 0.001]. IL28B-CC had 93.8% (211/225) D1L, while IL28B-CT/TT had 72.4% (284/392) D1L (p < 0.001). D1L was higher in HCV genotype 1 than in HCV genotype 4 [81.7% (425/520) *vs*. 73% (84/115); p < 0.05). However, neither mono-infected (p = 0.831) nor co-infected patients (p = 0.098) showed statistically significant differences when segregated with respect to HCV genotype. Cirrhotic patients had less D1L than non-cirrhotic patients [66.3% (67/101 *vs*. 82.4% (402/488); p < 0.001]. FIB-4, APRI and Forns’ index were significantly lower (p < 0.001) in patients with D1L. Significant fibrosis was related to less D1L [74.3% (205/276) *vs*. 84.5% (284/336); p < 0.005].

### Development of the prognostic tool (“Optim”) to predict D1L

**Fig 4:**
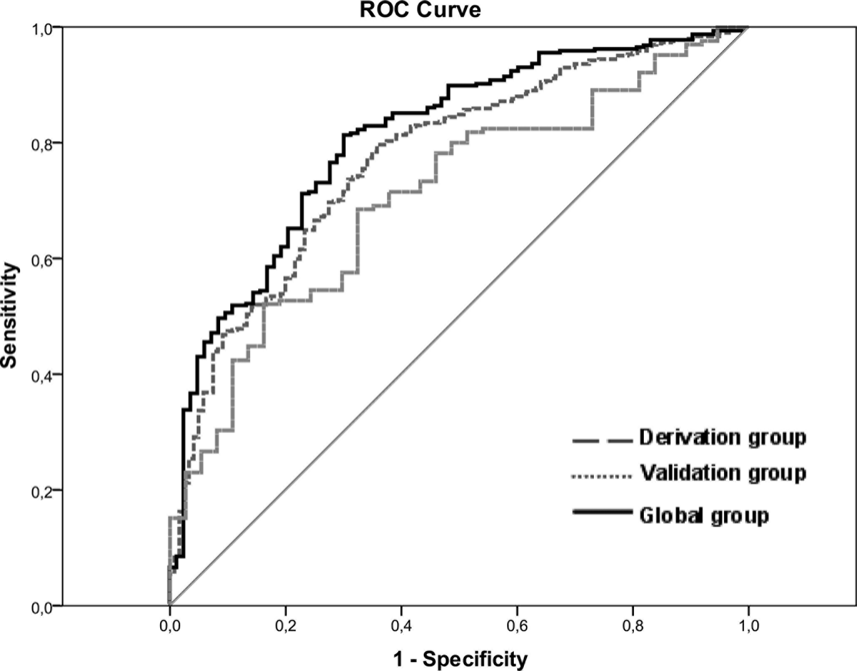
AUROC curve analysis for R_D1L_, including the overall cohort (0.79; p<0.001), the estimation cohort (0.77; p<0.001) and the validation cohort (0.71; p<0.001)

In multivariate analyses, variables associated with D1L were: LVL [OR: 1.88 (95%CI: 1.04–3.38); p = 0.035], HIV co-infection [OR: 0.49 (95%CI: 0.27–0.88); p = 0.016], IL28B-CC [OR: 8.75 (95%CI: 3.78–20.25); p < 0.001], Genotype 1 [OR: 1.93 (95%CI: 0.99–3.74); p = 0.05], Forns’ Index [OR: 0.73 (95%CI: 0.62–0.85); p < 0.001] (Table 4). The AUROC was 0.81 (95%CI: 0.76–0.86; p < 0.0001) and the cut-off 0.733 showed a sensitivity of 78%, specificity of 65%, positive predictive value of 90% and negative predictive value of 42%. This model was confirmed in the validation cohort, except for the HCV genotype, with an AUROC of 0.71 (95%CI: 0.62–0.79); p < 0.001) (Fig 4) and a cut-off 0.733 showing a sensitivity of 82%, specificity of 46%, positive predictive value of 82% and negative predictive value of 37%.

**Table 4:** Variables associated with D1L in multivariate analysis of the estimation cohort.

Characteristics	O.R. (95% CI)	P
Co-infected patients	0.49 (95%CI: 0.27–0.88)	0.016
HCV genotype 1	1.93 (95%CI: 0.99–3.74)	0.05
IL28B-CC polymorphism	8.75 (95%CI: 3.78–20.25)	< 0.001
Forns`index	0.73 (95%CI: 0.62–0.85)	< 0.001
Low viral load	1.88 (95%CI: 1.04–3.38)	0.035

The final formula was:

$${\text{R}}_{\text{D1L}}\text{= 1 / }\left({\text{1+e}}^{\text{-(2.909 +0.630 x HVL -0.719 co-infection+ 2.169 IL28B + 0.657 x genotype – 0.322 x Forns’ index)}}\right)\text{.}$$

Sensitivity of the model to predict SVR

SVR for RVR was 76.5% (101/132) and was 67% (146/218) for predicted RVR (p < 0.05). On the other hand, SVR for D1L was 51.7% (238/460) and was 54.3% (215/396) for predicted D1L (p > 0.05).

### Cost-analysis

Total cost for hepatitis C therapy per patient was estimated as €35,233 for the 48 weeks. The implementation of the proposed prognostic tool was associated with €10,665 saving/patient, in the base case scenario.

The total savings per patient in alternative scenarios ranged from €12,298 to €9,627 (Table 5). Assuming a fixed budget of €1,000,000, the implementation of the prognostic tool is to enable treatment of an additional 12 patients in the base case scenario.

**Table 5:** Cost-analysis of application of the “Optim” strategy (decimals rounded to the nearest euro).

		Implementation of prognostic tool		
		Yes	No	Difference
Total treatment cost for hepatitis C therapy (48 weeks) per patient	Base case scenario	€ 24,568	€ 35,233	€ -10,665
	Alternative scenario 1: Triple therapy cost = €36,352	€ 25,207	€ 36,352	€ -11,145
	Alternative scenario 2: Triple therapy cost = €34,144	€ 23,903	€ 34,114	€ -10,184
	Alternative scenario 3: Prognostic tool’s sensitivity (upper limit 95%CI) and RVR positive predictive value	€ 22,935	€ 35,233	€ -12,298
	Alternative scenario 4: RVR prognostic tool’s sensitivity (lower limit 95%CI) and RVR positive predictive value	€ 25,606	€ 35,233	€ -9,627

## Discussion

The combination of IL28B genotype with viral load, HCV-genotype, Forns’ Index (a non-invasive marker of fibrosis) and the presence of HIV co-infection enabled us to construct a tool able to predict virological response at week 4 in patients treated with peginterferon alfa-2a+ribavirin.

In the course of boceprevir research & development for the treatment of patients with chronic hepatitis C genotype 1, a lead-in phase over 4 weeks was included in the design of the majority of clinical trials. Theoretically, this approach was to improve sustained virological response rate [15] and lower the rate of emergence of resistant variants. Despite both end-points not having been confirmed, the lead-in phase with P+R appeared to have helped a better classification for interferon sensitivity. Patients with RVR showed the same rate of SVR when treated with double or triple therapy, while patients without a decline of 1 log HCVRNA at week 4 did not achieve SVR despite receiving triple therapy. As reported by Marcellin et al [16] in a cohort of 558 patients with hepatitis C genotype 1, those with hepatitis C genotype 1 achieving RVR showed a rate of SVR >85%. In a sub-analysis of the SPRINT-1 study [14], patients without D1L achieved SVR rate of <15%. Thus, using the 4-week virological response could be very useful in making decisions in the management of hepatitis C genotype 1. Also, considerable cost saving could be made as well as adverse events being pre-empted in patients with little or no chance of achieving a cure in non-D1L, or in patients who do not require triple therapy because of having achieved RVR.

**Fig 5:**
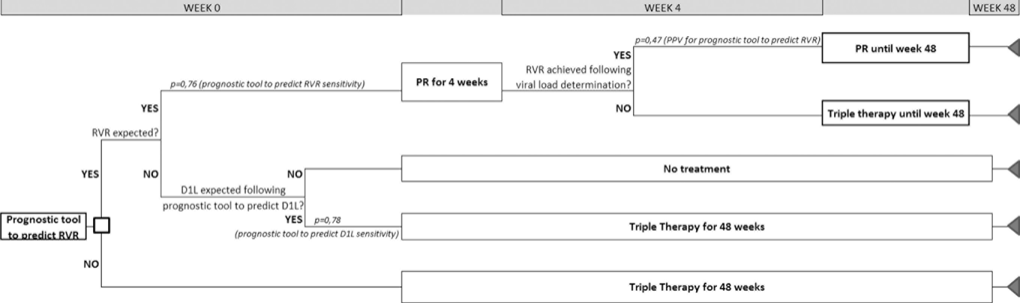
The decision tree based on sensitivity of the proposed prognostic tool to predict RVR and D1L.

The “Optim” tool, based on genetic, viral and host factors enabled us to predict treatment response at 4 weeks by mixing these 5 variables. Individualization of therapy appears to be crucial in improving the management of hepatitis C in clinical practice. Martinez-Bauer et al [17], combined baseline and week 4 virological response to predict SVR. Baseline viral load, AST/ALT ratio, serum cholesterol, and non-invasive estimation of liver fibrosis were included, together with RVR. Response was predicted accurately in approximately 60% of genotype 1 patients. A link between early viral dynamics and SVR has been well documented. In patients treated with P+R, the reduced SVR rates in patients >45 years of age, with severe liver fibrosis and high baseline viral load, were strongly associated with slower second phase decline of HCV-RNA [18]. These data were also confirmed in HIV/HCV co-infected patients [19]. Moreover, in a cohort of 113 HCV-genotype 1 patients, complete early virological response (cEVR) was the viral factor most strongly predictive of SVR in non-RVR patients [20]. Lastly, the combination of factors such as an evaluation of sequence of interferon-sensitivity-determining region (ISDR), T-helper 1/T-helper 2 ratio, body weight, and neutrophil count could predict SVR accurately at baseline [21]. Nevertheless, IL28B polymorphism needs to be added to this group of factors predicting virological response. Indeed, IL28B was found associated with RVR, EVR, ETR and SVR in treatment-naïve patients of HCV genotype 1 chronic infection [22], and was highlighted as the strongest factor predicting RVR in a large cohort of 1587 patients [23]. Thus, combining host and viral factors together with IL28B appears to build the most solid tool to predict D1L and RVR in patients undergoing P+R therapy. This observation at 4 weeks could preclude any further treatment over the standard scheduled 48 weeks. This tool would be freely available (http://www.optimtool.com/formula.html) for decision making regarding hepatitis C treatment and could confidently predict achieving D1L and RVR in patients with hepatitis C genotypes 1&4. This is possible irrespective of co-infection by HIV, or measurement of viral load, or the non-invasive estimation of fibrosis using the Forns’ Index. Although our study was designed to develop this tool in treatment-naïve patients, a similar approach could be explored in patients with previous treatment failure. Indeed, the Realize study demonstrated that, in the lead-in phase arm, SVR rate was lower in previously-treated non-responders without D1L. This supports the concept that, in these patients with functional mono-therapy, triple therapy should not be initiated since it is doomed to be ineffective or superfluous. The implementation of our predictive tool of viral response at week 4 could be useful in the assessment of those potential candidates for antiviral treatment (Fig 5). It enables the identification of a subgroup of patients having a low probability of achieving a reduction of HCV-RNA <1 log after 4 weeks of combination therapy (lead-in) and, in whom, the probability of SVR to triple therapy is suboptimal. It could be argued that, in these patients, the benefit of boceprevir- or telaprevir-based triple therapy is limited and, as such, it would be reasonable to await the advent of more effective agents that would pre-empt adverse events and allow a reallocation of available resources to those patients who have an increased likelihood of achieving SVR. In addition, this predictive tool could identify patients having a high probability of response to P+R (those with a high probability of achieving RVR) in whom dual or triple therapies are equally effective and, as such, the protease inhibitor may be best reserved for second-line therapeutic use. Further, this tool may help ensure that resources are used in an efficient manner since it would result, in a base-case scenario, in a reduction of 29.6% in the budget.

The results of the study may be particularly useful in determining when triple therapy could be the most efficient approach. It could be helpful, as well, in determining whether the therapy should even be initiated. Although the addition of telaprevir to P+R has clearly improved SVR rate for HCV genotype 1, adverse events and costs have emerged as relevant barriers for generalized use around the world. The median total cost for 48 weeks of protease inhibitor-based triple therapy was higher, including direct and indirect costs [24]. However, this cost analysis rests on the assumption that patients will be willing to be treated with dual therapy and, in some cases with a low probability of achieving D1L, to await new therapeutic developments. In spite of the recent approval of the new direct acting antivirals, therapy based on peginterferon and ribavirin will keep playing an important role in particular scenarios. In the most cost-sensitive countries, the reported tool could help to distinguish those patients for dual or triple therapy and, consequently, optimize the resources. The aim of the proposed strategy is to maximize benefits from the National Health Service (NHS) viewpoint since an NHS is charged with providing the greatest health-care benefits within the available resources. In this context, it is unclear how patients will respond to options that may not fulfill their therapeutic expectations.

Our cost-analysis has several limitations: a) we did not considered either response-guided therapy or futility rules of triple therapy in our model. This was because of lack of individual data regarding the relevance of D1L in short treatment regimens in the pivotal studies of boceprevir- and telaprevir-based treatments [25,26]. This approach may overestimate the cost savings in non-cirrhotic patients; b) since the treatment costs of the 2 protease inhibitors differ in different countries, we assumed an average cost of a general protease inhibitor treatment for 48 weeks. In assessing the consistency of the estimations made on the base case, alternative scenarios were tested and showed similar results. Our findings in the present study are limited to previously-untreated patients and should be interpreted only in the context of this population.

In conclusion, the combination of genetic (IL28B polymorphism) and viral genotype together with viral load, HIV co-infection and fibrosis stage defined a tool to predict RVR and D1L at week 4. The implementation of this tool in clinical practice could be a cost-saving strategy, compared to the universal triple therapy for hepatitis C. As such, it could contribute to a more efficient allocation of limited resources.
